# Analysis of risk factors for the development of type 2 diabetes mellitus complicated with Hashimoto’s thyroiditis

**DOI:** 10.1186/s12902-022-01092-6

**Published:** 2022-07-08

**Authors:** Meng Han, Haoneng Wu, Weiming Yang, Juanjuan Chen

**Affiliations:** 1 Jiangxi Province Key Laboratory of Laboratory Medicine, Department of Clinical Laboratory, the Second Affiliated Hospital of Nanchang University, Nanchang, 330006 Jiangxi P.R. China; 2 Queen Mary School, Nanchang University, Nanchang, 330006 Jiangxi P.R. China; 3 School of Public Health, Nanchang University, Nanchang, 330006 Jiangxi P.R. China

**Keywords:** Type 2 diabetes mellitus, Hashimoto’s thyroiditis, Thyroid-stimulating hormone, Risk factors, Glycosylated hemoglobin, Parathyroid hormone

## Abstract

**Aims:**

The purpose of this study is to elucidate the correlation between thyroid hormone, glycosylated hemoglobin (HbA1c), vitamin D and type 2 diabetes mellitus (T2DM) with Hashimoto’s thyroiditis (HT), and to seek the independent predictors affecting disease development.

**Methods:**

The study included 44 T2DM with HT, 94 T2DM, and 112 healthy subjects. We investigated some laboratory factors like thyroid hormone and compared the levels. Independent predictors determination by logistic univariate regression analysis were analyzed. The diagnostic value of thyroid-stimulating hormone (TSH) and threshold concentration were determined by ROC curve.

**Results:**

In T2DM with HT group, levels of PTH, HbA1c were lower and levels of TSH were significantly higher, when compared with T2DM group. But there was no significant difference in vitamin D between these two groups. In both logistic univariate regression analysis and multiple logistic regression analysis, TSH, HbA1c were independent predictors for T2DM with HT. Based on the ROC curve, the best cut-off value of the TSH was 4 mIU/L (sensitivity 72.7%, specificity 94.6%, AUC = 0.832) for predicting T2DM with HT in T2DM patients.

**Conclusions:**

TSH has increased risk for T2DM evolving into T2DM complicated with HT, so it is important to monitor the concentrations of TSH in patients with T2DM. Although vitamin D was not the independent predictor in T2DM with HT development, effect of vitamin D deficiency on the progress of diabetes and its complications should be taken into consideration.

## Background

Hashimoto’s thyroiditis (HT) is an autoimmune thyroid disease (AITD) that induces chronic inflammation of the thyroid tissue and thyrocyte destruction, companying with hypothyroidism in almost 20%–30% of patients [1, 2]. It is a classical T cell-mediated autoimmune disease with predominant infiltration of CD4^+^ type 1 T helper (Th1) subtype into the thyroid gland as major characteristics [3]. 25(OH)D is the circulated and deposited form of vitamin D, and this form is considered as the best indicator to measure whole vitamin D at serum levels. Interestingly, vitamin D receptors (VDRs) have been discovered in almost all immune cells, including activated CD4^+^ and CD8^+^ which give us the awareness for vitamin D functions in the regulation of immune response [4]. As research progressed, vitamin D receptors were found not only in immune cells but also in pancreatic β cell [5, 6]. Stimulation of β cells by an activated form of vitamin D can increase insulin secretion [6]. Hence, vitamin D deficiency is associated with a decreased insulin release, inducing insulin resistance which leads to type 2 diabetes mellitus (T2DM) [6].

In addition, the correlation between DM and thyroid dysfunction was stated as early as 1979 [7, 8]. The prevalence of thyroid dysfunction in diabetes are range from 2.2% to 46.5% [7–11]. Many researches have demonstrated that thyroid dysfunction is more prevalent in T2DM patients compared with normal population [12, 13]. Moreover, the most common thyroid dysfunction in T2DM patients was subclinical hypothyroidism which is usually with high thyroid-stimulating hormone (TSH) levels and normal free triiodothyronine (FT3), free thyroxin (FT4) levels [7, 10, 12]. TSH, one of important hormones in hypothalamic-pituitary-thyroid axis, serves as a master regulator of thyroid functions, affecting almost all cellular process of thyroid hormone production [14, 15]. With the development of research, we find that TSH regulates many metabolic process, such as glucose metabolism. A study showed that higher TSH levels were related with a higher risk of diabetes [16]. Furthermore, TSH also plays a vital role on immune system, especially for lymphocyte homeostasis, which gives us a speculation for its involvement in AITD like HT [17].

Therefore, based on previous studies, the T2DM with HT patients have already become important parts of T2DM patients. Most researches have only focused on the relationship between type 2 diabetes or the relationship between HT with thyroid hormone and vitamin D independently. Therefore, tying the two diseases together to see the combination effects seems left behind, which leads us to focus on the patients of T2DM with HT. The purpose of this study was to explore the related factors like thyroid hormone, vitamin D, parathyroid hormone (PTH), HbAc1 in T2DM with HT patients, finding out the possible factors for disease development that are important in further prevention.

## Methods

### Patients

Data were attained from subjects who have hospitalized and outpatient in Second Affiliated Hospital of Nanchang University from January 2014 to July 2019. Inclusion criteria: adults; subjects who underwent level tests of thyroid function, HbAc1, PTH and 25(OH)D levels in laboratory. Exclusion criteria: (1) under the age of 18 (2) contain other autoimmune diseases in addition to Hashimoto’s thyroiditis (3) patients with type 1 diabetes mellitus (4) patients with obvious liver, kidney, lung, heart function insufficiency (5) patients with benign or malignant tumors; (6) pregnant and lactation patients (7) patients with other thyroid diseases not caused by HT (8) patients suffering from other bone metabolic diseases.

### Diagnostic criteria

The plasma glycose in oral glucose tolerance test higher than 200 mg/dL characterized as T2DM [18]. Patients who have higher levels of anti-thyroid peroxidase antibody (TPO-Ab) than 35 IU/mL or anti-thyroglobulin antibody (TG-Ab) than 40 IU/mL and thyroid ultrasound evaluation with diffuse enlargement of the thyroid gland are regarded as T2DM with HT patients [19]. According to the Endocrine Society guidelines, the level of 25(OH)D below 20 ng/mL is deficiency (*N* = 25), and that above 20 ng/mL is non-deficiency (*N* = 19) [19]. Normal range was 0.55 to 4.78 mIU/L for TSH, 2.3 to 4.2 pg/mL for FT3, and 0.89 to 1.8 ng/dL for FT4 by chemiluminescence immunoassay.

### Clinical and laboratory test with equipment

The laboratory test was carried out after the diagnosis. 3 mL peripheral venous blood was collected for all studied subjects following centrifugation for 2500 rpm/min, 15 min. The test of PTH and 25(OH)D levels was measured by Cobas e601 automatic electrochemiluminescence immunoassay instrument (Roche, Switzerland) with chemiluminescence methods. TSH levels were detected by ADVIA Centaur XP fully-automatic chemical luminescence immunity analyzer (Siemens, Germany). We Used the VARIANT II Glycated Hemoglobin HbA1c Meter (Bio-Rad, USA) by high performance liquid chromatography (HPLC) method to test HbA1c level. All operations were strictly in accordance with the instructions of reagent and the standard quality management rules of the Second Affiliated Hospital of Nanchang University.

### Statistical analysis

The power was calculated at level sample size 138, by entering the following parameters: alpha = 0.05, N (Sample Size) = 138, P0 (Baseline Probability that Y = 1) = 0.2, 0.25, 0.3, 0.4, 0.5, Odds Ratio (Odds1/Odds0) = 2.0, R-Squared of X1 with Other X’s = 0.27, X1 (Independent Variable of Interest) = Continuous (Normal). The PASS 15.0 software calculated the power automatically as the following: 0.79 (P0 = 0.2), 0.85 (P0 = 0.25), 0.88 (P0 = 0.30), 0.92 (P0 = 0.40), 0.93 (P0 = 0.50).

All statistical analysis was carried out by SPSS17.0 for Windows. The mean value was expressed as mean ± SD in continuous data. Kolmogorov-Smirnov was carried out for normality test. Comparisons of continuous variables within three groups were performed by Kruskal-Wallis test. The statistically significant variables in univariate and multiple logistic regression analysis were defined as independent predictors of the presence of T2DM with HT. *P* < 0.05 were regarded as statistically significant. In the logic regression, the T2DM with HT patients and T2DM patients were defined as dependent variable. Sex and age were regarded as covariates in logistic regression model.

## Results

### General data of the subjects

There were 250 subjects from January 2014 to July 2019 in total, including 44 examinees of type 2 diabetes with Hashimoto’s thyroiditis, 94 examinees of type 2 diabetes and 112 healthy examinees. General data like age, sex are shown in Table 1. There are no statistically significant difference between three groups in terms of age (*P* = 0.277) and sex (*P* = 0.06).

**Table 1:** General data in the groups of type 2 diabetes with Hashimoto’s thyroiditis, type 2 diabetes and healthy control

Characteristics	T2DM with HT (*N* = 44)	T2DM (*N* = 94)	Healthy (*N* = 112)	*P*-value
Male	11	30	47	0.060
Female	33	64	65	
Age, years	57.00 ± 10.20	59.13 ± 10.57	59.13 ± 10.57	0.277

Data were analyzed using Kruskal-Wallis test

### Comparison between T2DM with HT patients and T2DM patients under the basis of related factors

As shown in Table 2, there were significant differences of 25(OH)D levels in the T2DM group (*P* < 0.05) and T2DM with HT group (*P* < 0.05) compared with the healthy group respectively, but there was no difference between the latter two groups (*P* > 0.05). There were significant differences in levels of PTH (*P* < 0.001), HbA1c (*P* < 0.001) and TSH (*P* < 0.001) when comparing the T2DM group and T2DM with HT group. The concentrations of TSH were higher in T2DM with HT (10.81 ± 18.98) than those in T2DM patients (2.05 ± 1.69). However, reversely the HbA1c levels (9.30 ± 2.45) were higher in T2DM patients.

**Table 2:** Comparison between T2DM with HT patients, T2DM patients and healthy people

	T2DM with HT	T2DM	Healthy
25(OH)D (ng/mL)	19.96 ± 7.82^**b**^	21.68 ± 6.39^**b**^	30.96 ± 5.55
PTH (pg/mL)	41.27 ± 15.16^**a**^	55.71 ± 23.98^b^	43.41 ± 14.02
HbA1c (%)	7.4 ± 1.66^**ab**^	9.30 ± 2.45^b^	5.33 ± 0.46
TSH (mIU/L)	10.81 ± 18.98^**ab**^	2.05 ± 1.69	3.23 ± 3.66
FT3 (pg/mL)	3.21 ± 1.59^b^	2.89 ± 0.45^b^	3.07 ± 0.34
FT4 (ng/dL)	1.19 ± 0.39^**b**^	1.28 ± 0.26	1.28 ± 0.21

Data are presented as mean ± SD; Data were analyzed using Kruskal-Wallis test

^a^Two-sided *P*-value is statistical significant in T2DM with HT group compared with T2DM group, *P* < 0.05

^b^Two-sided *P*-value is statistical significant when compared with healthy group respectively, *P* < 0.05

### Independent predictor analysis of type 2 diabetes with HT

In both logistic univariate and multiple regression analysis (Table 3), T2DM with HT patients were correlated with TSH and HbA1c. Both PTH (OR 0.934, 95% CI 0.878–0.994, *P* = 0.076) and 25(OH)D more than 20 ng/ml (OR 0.285, 95% CI 0.055–1.472, *P* = 0.134) was not statistically significantly correlated under the influence of multiple factors in type 2 diabetes with HT.

**Table 3:** Logistic regression analysis of T2DM with HT prevalence by 25(OH)D, HbA1c and thyroid function factors

	Univariate		Multivariate	
Exposure	OR (95%CI)	*P*-value	OR (95%CI)	*P*-value
25(OH)D more than 20 ng/ml	0.470 (0.227–0.973)	0.042	0.285 (0.055–1.472)	0.134
PTH	0.947 (0.919–0.975)	< 0.001	0.934 (0.878–0.994)	0.076
HbA1c	0.630 (0.501–0.792)	< 0.001	0.575 (0.351–0.944)	0.029
TSH	1.665 (1.313–2.110)	< 0.001	2.002 (1.270–3.155)	0.007
FT3	1.570 (0.787–3.134)	0.201	21.102 (2.3131–192.548)	0.201
FT4	0.394 (0.99–1.564)	0.185	0.201 (0.014–2.827)	0.458

Sex and age were regarded as covariates in logistic regression model

### Using receiver operator characteristic curve to evaluate the diagnostic value of TSH for T2DM with HT patients and to determine threshold concentration

A receiver operator characteristic (ROC) curve was used to carry out the best threshold for concentrations of TSH to distinguish the T2DM and T2DM with HT.

**Fig. 1:**
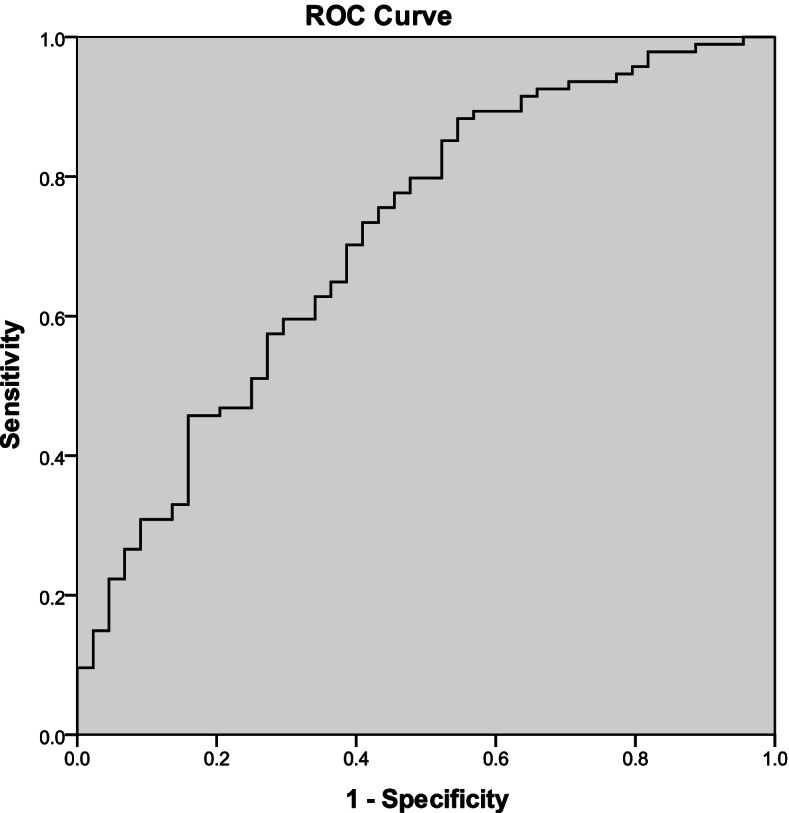
The identification of TSH threshold values in ROC curve. ROC curve used to determine the TSH threshold values in T2DM patients with HT. The threshold value is 4 mIU/L in TSH concentration, sensitivity was 72.7%, specificity was 94.6%

We found the threshold concentration of TSH of 4 mIU/L, with specificity of 94.6% and sensitivity of 72.7% (AUC = 0.832; Fig. 1).

## Discussion

Thyroid dysfunction has been reported to be associated with T2DM in many studies [20, 21]. Some studies have demonstrated a bidirectional impact of diabetes and thyroid disorders upon each other [7, 12, 22]. Our study found significant differences in TSH between T2DM group and T2DM with HT group. Thyroid hormones were regarded as key factors which could monitor the HT development in T2DM patients in our study. Some researchers believe that about 8% of women and 3% of men have subclinical hypothyroidism in the patients who have asymptomatic chronic autoimmune thyroiditis [23]. Moreover, HT has exerted a negative impact on the subclinical hypothyroidism progression overtime. In one study, the comparison between HT-related subclinical hypothyroidism and idiopathic subclinical hypothyroidism demonstrated the higher risk of a thyroid statue deterioration and lower probability of a spontaneous TSH normalization in HT-related subclinical hypothyroidism [21]. Based on this study, we can speculate that HT-related subclinical hypothyroidism patients are likely to remain in subclinical hypothyroidism state for a long time. Other studies have shown that the elderly patients with high antithyroid antibody may have higher risk for overt hypothyroidism development [24]. However, in fact, whether patients with HT-related subclinical remain in subclinical state or further develop into overt hypothyroidism is actually affected by many factors. In T2DM patients, the incidence of thyroid disorders especially for subclinical hypothyroidism was found to be 26.7% [25]. The mechanisms involved have not been clearly studied, but have been shown poor glycemic control was associated with the risk of subclinical hypothyroidism in T2DM patients [26]. Based on pervious study, the occurrence of subclinical hypothyroidism is very common in both HT patients and T2DM patients independently. Therefore, the speculation can be made for the common occurrence of subclinical hypothyroidism in T2DM with HT patients, which is consistent with our statistical results. In our study, we found that TSH was significantly increased in T2DM with HT, while no significant in FT3 and FT4, suggesting that T2DM with HT patients were more predisposed to subclinical hypothyroidism. Although TSH is a master regulator of thyroid functions, its function in immune system and glucose metabolism still need to be taken into consideration. Previous research has identified the crucial role of TSH on lymphocyte homeostasis especially for T-cell number and cell population [17, 27]. In congenital hypothyroidism mouse model with high level of TSH, one study demonstrated the lower CD8^+^ percentage and higher CD4^+^ percentage in thymocytes compared with euthyroid mice [17, 28]. This led us to associate TSH with HT, which is characterized by CD4^+^ cell infiltration. Moreover, thyroid hormone has been demonstrated to regulate glucose metabolism and pancreatic function [29]. One study showed higher TSH level with a higher possibility of diabetes [16]. The possible pathogenesis can be explained by the direct induction of thyroid hormone to HIF-α through PI3K/ERK pathway with multiple factors which regulate glucose metabolism [29]. Based on previous studies about HT or T2DM independently, speculation of TSH and T2DM with HT patients can be made as its central role in metabolic regulation and immune system regulation. Therefore, we emphasize that the TSH level is a predictor of T2DM with HT in T2DM patients, especially at the concentration exceed 4.05 mIU/L.

In addition to thyroid hormone, our study found that there were significant differences in HbA1c between T2DM group and T2DM with HT group. The level of HbA1c in T2DM group was higher than those in T2DM with HT group. This result may be explained by the fact that HbA1c level reflects the average level of blood sugar of patients for two to 3 months, as diabetic patients usually result in hyperglycemia, so controlling the normal level of HbA1c is important for T2DM development [30]. Both univariate regression analysis and multiple regression analysis demonstrated the correlation in HbAc1 group. Hence, HbA1c was an independent predictor of T2DM with HT patients. Some researchers demonstrated that thyroid dysfunction increased with the rise of HbA1c. They suggest that poor glycemic control may be associated with thyroid dysfunction development in T2DM patients [11, 31]. However, this differs from the findings presented here. The HbAc1 level in T2DM with HT group was higher than that in health group, but was not higher than that in the T2DM group, which was inconsistent of the result with the higher HbA1c in thyroid dysfunction with T2DM patients in some previous studies [11, 32]. However, this discrepancy could be attributed to thyroid hormone function. As the thyroid hormone plays an important role in stimulating erythropoiesis, increasing erythropoietin production and effecting on iron utilization. Bremner et al. has demonstrated the impaired ion utilization in subclinical hypothyroidism usually company with erythropoiesis reduction [33]. Furthermore, the subclinical hypothyroidism subjects treated with thyroid hormone replacement have experienced a decrease in HbA1c levels due to increased erythropoiesis rather than a change in glucose levels [34]. This indicated that the HbAc1 level in subclinical hypothyroidism patients is not only correlating with glycose control, but also correlating with the erythropoiesis process. Although the HbA1c is independent predictor for T2DM with HT, the elevation or reduction may be due to the dual effect of T2DM situation and thyroid hormone function, which may various in different patients. Hence, the relationship between thyroid disease combined with T2DM and HbA1c needs further exploration.

Vitamin D is involved in both the immune system and sugar metabolism [4, 6]. In our study, there were significant differences in 25(OH)D levels when comparing the T2DM group with healthy group. This result supports the idea of vitamin D plays an important role in type 2 diabetes development that affect β cell function, insulin sensitivity, and the influence of systemic inflammation [35]. In addition to its role in T2DM, recent studies have shown that low vitamin D levels are also related to AITD like HT [36, 37]. Consistent with the literature, our study also showed significant differences in 25(OH)D levels between the T2DM with HT group and the healthy group. Based on previous studies about HT or T2DM independently, speculation of vitamin D and T2DM with HT patients can be made due to the both alteration in T-cell when low vitamin D state. However, the findings in our study did not support the previous researches. In our study, we referenced the endocrine society guidelines, classified 25(OH)D into two groups, those 25-hydroxyvitamin D[25(OH)D] levels < 20 ng/mL were classified as a vitamin D deficiency, and those > 20 ng/mL, as a non-deficiency group [38]. Nevertheless, correlation between T2DM with HT patients and 25(OH)D non-deficiency group was only shown in univariate regression analysis rather than in multivariate regression analysis, which indicated the pervious differences appeared in univariate regression analysis might be caused by other factors. Although all of the mechanisms above have provided evidences for the relationship of vitamin D in HT patients or T2DM patients and make some possible speculation for the mechanism in T2DM with HT, it is not enough to set up the significant linkage between vitamin D and T2DM with HT patients, which still needs further consideration.

PTH is secreted by the parathyroid gland as a master regulator of calcium, phosphate metabolism. Our study suggested the significant differences in PTH levels when comparing the T2DM group and T2DM with HT group. However, the statistical significance for PTH was only observed in univariate regression analysis rather than in multiple regression analysis, which indicated that there were differences between these two groups but no disease risk changing in PTH involvement.

The present study has some limitations, for example the sample size for T2DM with HT is not relatively large enough. However, it is possible to gain significant results and find some possible correlation between T2DM and T2DM with HT patients.

## Conclusions

We emphasize the importance of regular monitoring the concentrations of TSH in patients with T2DM, as T2DM patients who develop with HT are usually in subclinical hypothyroidism states. The specific role of HbA1c in T2DM with HT patients remains to be further investigated, as the changes in HbA1c levels may be due to the dual effects of T2DM status and thyroid hormone function. TSH has increased the risk for T2DM evolving into T2DM complicated with HT. Greater attention should be paid to T2DM patients who have higher TSH with a view to prevent the complication of HT in early stage. Although there is no significant disease risk increase for vitamin D and PTH, the continuous consideration are still needed as they are both important factors for T2DM or HT independently.

## Data Availability

The datasets used and/or analysed during the current study are available from the corresponding author on reasonable request.

## Abbreviations

T2DM: Type 2 diabetes mellitus
HT: Hashimoto’s thyroiditis
HbA1c: Glycosylated hemoglobin
TSH: Thyroid-stimulating hormone
PTH: Parathyroid hormone
FT3: Free triiodothyronine
FT4: Free thyroxin
AITD: Autoimmune thyroid disease
